# A solid pseudopapillary neoplasm without cysts that occurred in a patient diagnosed by endoscopic ultrasound-guided fine-needle aspiration: a case report

**DOI:** 10.1186/1752-1947-8-243

**Published:** 2014-07-03

**Authors:** Masakuni Fujii, Masao Yoshioka, Takefumi Niguma, Hiroaki Saito, Toru Kojima, Soichiro Nose, Junji Shiode

**Affiliations:** 1 Department of Internal Medicine, Okayama Saiseikai General Hospital, 1-17-18 Ifuku-cho, Okayama 700-8511, Japan; 2 Department of Surgery, Okayama Saiseikai General Hospital, 1-17-18 Ifuku-cho, Okayama 700-8511, Japan; 3 Anatomic Pathology, Okayama Saiseikai General Hospital, 1-17-18 Ifuku-cho, Okayama 700-8511, Japan

**Keywords:** Atypical, Endoscopic ultrasound-guided fine-needle aspiration, Minimally invasive surgery, Positron emission tomography/computed tomography, Solid pseudopapillary neoplasm

## Abstract

**Introduction:**

Solid pseudopapillary neoplasm of the pancreas is a rare neoplasm that has been reported to account for between 0.17% and 2.7% of all non-endocrine tumors of the pancreas. It is usually seen in young women. Because solid pseudopapillary neoplasms are rarely aggressive and have low-grade malignant potential and an excellent prognosis after complete resection, it is an ideal pancreatic tumor for treatment by minimally invasive surgery. Therefore, making an accurate pre-operative diagnosis is very important.

**Case presentation:**

A 24-year-old Japanese man who had been found to have mild transaminase elevations at a medical check-up visited our hospital for further examination. Abdominal computed tomography showed a 40mm-diameter tumor in the pancreatic tail and mild fatty liver. He was admitted to our hospital for additional examination. The abdominal contrast-enhanced computed tomography scan taken at our institution showed an increasingly enhanced mass of 40mm diameter in the pancreatic tail. Ultrasonography showed a low-level echoic mass of 35mm diameter in the pancreatic tail. T1-weighted magnetic resonance imaging showed low signal intensity in the tail of the pancreas. T2-weighted magnetic resonance imaging showed high signal intensity there. Diffusion magnetic resonance imaging showed high signal intensity. An endoscopic ultrasound yielded the same results as the abdominal ultrasonogram. In addition, [^18^F]-fluorodeoxyglucose positron emission tomography/computed tomography showed abnormal accumulation (maximum standardized uptake value, 6.53). This finding raised our suspicion of a pancreatic malignant tumor. However, the patient could not be confidently diagnosed solely on the basis of imaging. Endoscopic ultrasound-guided fine-needle aspiration was performed, which led us to a diagnosis of solid pseudopapillary neoplasm. On that basis, we performed minimally invasive surgery (spleen-preserving laparoscopic distal pancreatectomy).

**Conclusion:**

Atypical solid pseudopapillary neoplasm without cysts should be considered when diagnosing pancreatic tumors. A definitive pre-operative diagnosis of solid pseudopapillary neoplasm made on the basis of endoscopic ultrasound-guided fine-needle aspiration can guide the surgical approach used.

## Introduction

Solid pseudopapillary neoplasm (SPN) of the pancreas is a rare neoplasm that has been reported to account for between 0.17% and 2.7% of all non-endocrine tumors of the pancreas. It is usually seen in young women and is usually asymptomatic. The lesion is generally large (2.5cm to 10cm) and encapsulated and frequently contains varying amounts of necrosis, hemorrhage, calcification and cystic changes [1]. Because SPN is rarely aggressive, has low-grade malignant potential and carries an excellent prognosis after complete resection, it should be differentiated from other, more aggressive tumors, such as adenocarcinoma and endocrine tumors [2]. SPN is an ideal pancreatic tumor for treatment by minimally invasive surgery. Therefore, pre-operative accurate diagnosis is very important. Endoscopic ultrasound (EUS)-guided fine-needle aspiration (EUS-FNA) has recently been established as a modality for use in the diagnosis of pancreatic mass-related lesions.

We present a case of a patient with atypical SPN that was diagnosed by performing EUS-FNA before surgery. We were able to perform minimally invasive surgery (a spleen-preserving laparoscopic distal pancreatectomy). Our findings suggest that a definitive pre-operative diagnosis of SPN made on the basis of EUS-FNA findings could guide the choice of surgical approach.

## Case presentation

**Figure 1:**
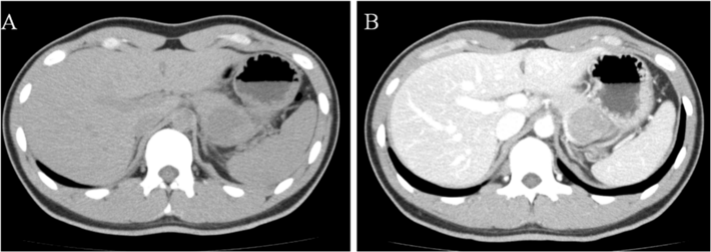
**Abdominal computed tomography showing the solid pseudopapillary neoplasm in our patient. (A)** This is a plain image, and it shows a mass of 40mm diameter in the pancreatic tail. **(B)** This is a contrast-enhanced image, and it shows an increasingly enhanced mass of 40mm diameter in the pancreatic tail.

**Figure 2:**
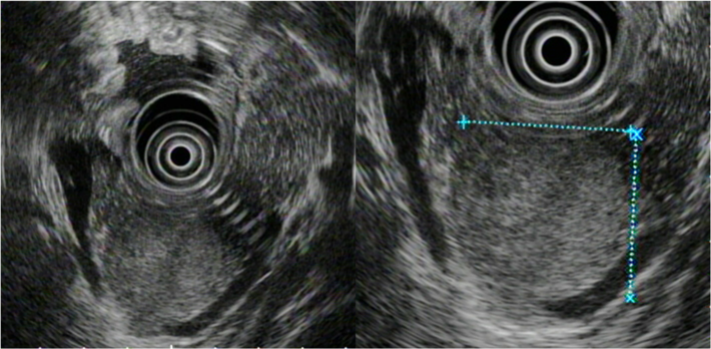
**Endoscopic ultrasound.** These two images show a low echoic mass of 35mm diameter in the pancreatic tail. (The left image shows a whole image and the right image shows an enlarged image. The dotted lines show lines of measurement for this tumor.)

**Figure 3:**
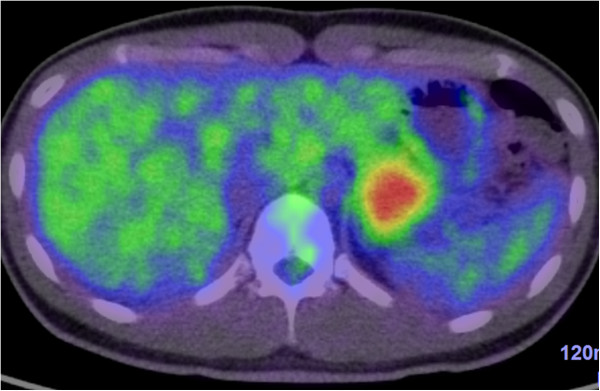
^**18**^**F-fluorodeoxyglucose positron emission tomography/computed tomography scans of the solid pseudopapillary neoplasm in our patient.** This fused image shows abnormal accumulation in the mass (maximum standardized uptake value, 6.53).

**Figure 4:**
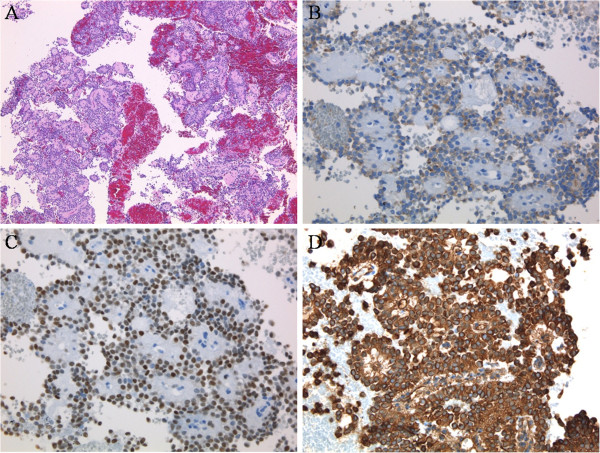
**Histopathological images of samples taken during endoscopic ultrasound-guided fine-needle aspiration. (A)** The tumor cells with round nuclei show pseudopapillary growth (hematoxylin and eosin stain; original magnification, ×4). Immunohistochemically, the tumor cells stained positive for CD10 (original magnification, ×10) **(B)**, progesterone receptor (original magnification, ×10) **(C)** and vimentin (original magnification, ×10) **(D)**.

**Figure 5:**
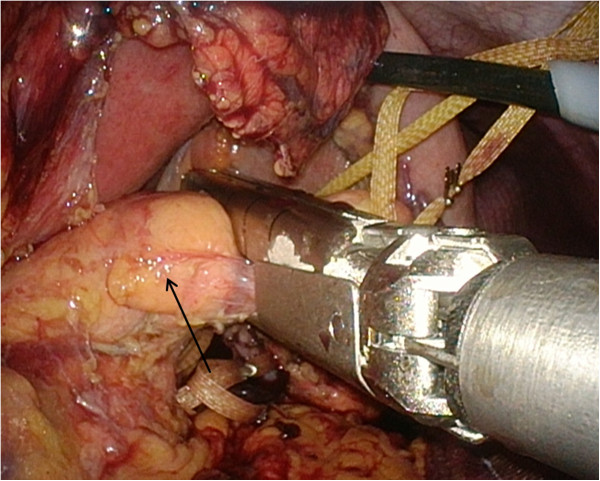
**Peri-operative photograph showing the pancreas.** Our patient was treated with a spleen-preserving laparoscopic distal pancreatectomy. The pancreas (arrow) was transected using a 60mm endoscopic gastrointestinal anastomosis stapler.

**Figure 6:**
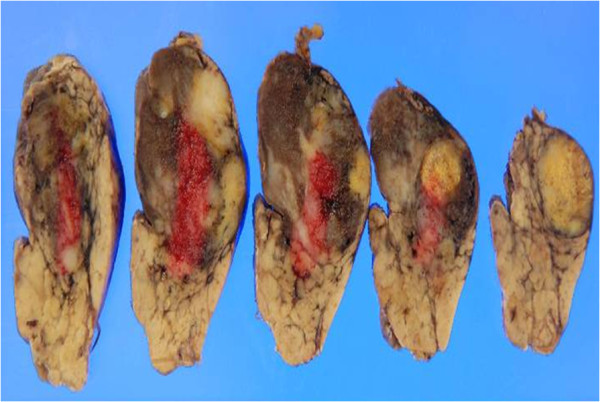
**Macroscopic images of the specimen.** Our examination of the resected specimen revealed a solid tumor extending to the tail of the pancreas. The tumor did not contain calcified or cystic areas.

A 24-year-old Japanese man was referred to our hospital after a medical check-up revealed mild transaminase elevations. He had no symptoms. He was found to have mild fatty liver and a pancreatic mass by contrast-enhanced computed tomography (CT). Abdominal contrast-enhanced CT showed an increasingly enhanced mass of 40mm diameter in the pancreatic tail (Figure 1). Ultrasonography (US) showed a low echoic mass of 35mm diameter in that location. T1-weighted magnetic resonance imaging (MRI) revealed low signal intensity in the tail of the pancreas, whereas T2-weighted MRI showed high signal intensity. Diffusion MRI showed high signal intensity. Our EUS (GF-UM 2000; Olympus Co, Tokyo, Japan) observations led us to the same conclusions as the abdominal US (Figure 2). In addition, [^18^F]-fluorodeoxyglucose positron emission tomography/computed tomography (FDG PET/CT) showed abnormal accumulation (maximum standardized uptake value, 6.53) (Figure 3), and this finding raised our suspicion of a malignant pancreatic tumor. However, the case could not be confidently diagnosed solely on the imaging results. FNA was therefore performed via a transgastric approach with linear EUS (GF-UCT 2000; Olympus Co), and two passes were made with a 25-gauge needle (EchoTip Ultra(ECHO-25); Cook Endoscopy, Winston-Salem, NC, USA). We noted no signs or symptoms in the patient during or after the procedure. Our histopathological findings derived from the samples taken during the EUS-FNA procedure included the presence of cells with round nuclei that showed pseudopapillary growth. The tumor cells showed immunopositivity for CD10, progesterone receptor and vimentin (Figure 4). We diagnosed this tumor as a SPN and performed minimally invasive surgery to resect it (a spleen-preserving laparoscopic distal pancreatectomy) (Figure 5). Upon examination of the resected specimen, we determined that it was a solid tumor extending to the tail of the pancreas. The tumor did not contain calcified or cystic areas (Figure 6). Our histopathological findings were similar to those obtained by EUS-FNA. The tumor cells showed immunopositivity for β-catenin, CD10 and vimentin. Our final pathological diagnosis was SPN of the pancreatic tail. No vascular invasion or infiltrative growth was observed. The tumor margin was negative. The patient’s post-operative course was good, and he had no post-operative recurrences in the 18-month follow-up period, during which time he was followed in the outpatient department.

## Discussion

SPN is an exceedingly rare pancreatic tumor with a reported frequency of less than 1% of all pancreatic diseases. In most cases (about 80%), patients with SPNs present either with pain or with a mass. The tumor is asymptomatic in about 15% of the cases [2]. Apostolidis *et al*. reported a very rare case in which hematemesis was a presentation of SPN [3]. SPN occurs predominantly in adolescent girls and young women. The occurrence of SPN in men is rare, accounting only for 7% of cases [4].

Our patient, a 24-year-old man, was asymptomatic. SPNs are generally large, with a mean diameter of 10.3cm. Approximately 72% of them arise in the body and tail of the pancreas. They less frequently develop in the head. In our patient, the tumor was comparatively large (40mm in diameter) and located in the tail of the pancreas. SPNs are generally encapsulated and frequently contain varying amounts of necrosis, hemorrhage, calcification and cystic changes [1]. However, our patient’s tumor had none of these characteristics. In addition, a previous case of small SPN with no cyst has been reported, suggesting that the cyst formation, bleeding, and calcification are secondary changes caused by tumor growth.

Because SPNs are of unclear pathogenesis and low malignancy and surgical resection offers the patient an excellent chance for long-term survival, these tumors should be differentiated from other, more aggressive tumors, such as adenocarcinoma and endocrine tumors [2].

The tumor characteristics on the images shown herein have a contrast enhancement, but this enhancement is weaker than it would be for a neuroendocrine tumor. The T1-weighted MRI shows low signal intensity, whereas the T2-weighted MRI shows high signal intensity. Although few articles about PET findings of SPN have been published to date, the frequency of these reports has increased slightly in recent years. Dong *et al*. described PET scans that showed high accumulation throughout most of the SPN. They postulated that the FDG uptake of SPN they observed may have been related to tumor cellularity, the proliferative index or histological malignancy [5]. Accordingly, SPN should be included in the differential diagnosis for pancreatic tumors with high accumulation in PET.

Most of the characteristics of our patient’s tumor were atypical of a SPN, so we could not diagnose it solely on the basis of imaging findings. We therefore performed EUS-FNA to obtain a more definitive diagnosis. The histologically based differential diagnosis of SPN from other malignant pancreatic tumors is very important, because SPN carries a much better prognosis than the other malignant pancreatic tumors, with only 10% to 15% of tumors recurring or metastasizing. More than 95% of SPNs are cured by complete surgical resection alone. In addition, it has been reported that minimally invasive surgery for SPN can achieve a favorable curative effect because of the non-aggressive behavior of the tumor, the presence of a dense capsule and the excellent prognosis. Marinis *et al*. reported a case of a patient with a SPN who was treated with laparoscopic distal pancreatectomy and concluded that SPNs are the ideal pancreatic tumors to be treated via the laparoscopic approach [6]. However, it is necessary to limit the removal of tissue to preserve as much normal pancreatic tissue as possible and to maintain its functional structure, because the tumor is usually found in young patients; therefore, it is important to consider these patients’ post-operative quality of life [7]. Accurate pre-operative diagnosis of SPN enables treatment with minimally invasive surgery. From this standpoint, the significance of the pre-operative pathological diagnosis cannot be overstated.

Recently, EUS-FNA has become a useful diagnostic and staging tool for patients with pancreatic tumors [8]. The sensitivity and specificity of EUS-FNA for pancreatic neoplasms have been reported to be 91% and 94%, respectively. The overall complication rate of EUS-FNA has been reported to be less than 1% in large centers. From among the 1,034 patients who underwent pancreatic EUS-FNA described in a previous report, complications consisted of only 10 patients (0.96%) with hemorrhage, 2 (0.19%) with acute pancreatitis and 1 (0.09%) with duodenal perforation [9].

Since Nadler *et al*. first described a correct diagnosis of SPN on the basis of EUS-FNA in 2002 [10], several SPN cases in which the diagnosis was made using this method have been reported [11]. Song *et al*. summarized the cytologic features in the 43 cases of SPN diagnosed on the basis of EUS-FNA described in the English literature. They reported that the cytomorphologic features observed after FNA are highly characteristic and distinct from those of other cystic or solid tumors of the pancreas [11]. Also, the diagnostic accuracy of EUS-FNA for SPN was found to be 75% in another study [12]. Minimally invasive surgery was performed in 29% of these patients. In addition, it has been reported that the diagnosis of SPN based on pre-operative EUS-FNA is feasible.

## Conclusion

We report a case of a 24-year-old man with pancreatic SPN without cysts. Other malignant pancreatic tumors were considered in the differential diagnosis, but we performed EUS-FNA to obtain a definitive diagnosis and were able to perform minimally invasive surgery. SPN without cysts should be considered when diagnosing pancreatic tumors. A definitive pre-operative diagnosis of SPN by performing EUS-FNA can guide the surgical approach.

## Consent

Written informed consent was obtained from the patient for publication of this case report and any accompanying images. A copy of the written consent is available for review by the Editor-in-Chief of this journal.

## Abbreviations

CT: Computed tomography; EUS-FNA: Endoscopic ultrasound-guided fine-needle aspiration; FDG PET/CT: Fluorodeoxyglucose positron emission tomography/computed tomography; MR: Magnetic resonance; SPN: Solid pseudopapillary neoplasm; US: Ultrasonography.

## Competing interests

The authors declare that they have no competing interests.

## Authors’ contributions

All authors were involved in the care of the patient. MF, MY and JS were the major contributors to the writing of the manuscript. MF and HS performed the EUS-FNA. SN made the pathological diagnosis. TK and TN performed the operation. TK has followed up this patient as an outpatient. All authors read and approved the final manuscript.
